# Unexpected Heartbreak: A Case Report of Takotsubo Cardiomyopathy Triggered by Electroconvulsive Therapy

**DOI:** 10.1155/cric/8140289

**Published:** 2025-10-24

**Authors:** Faeghe Hosseini, Amir Ghaffari Jolfayi, Mehdi Sheibani

**Affiliations:** ^1^Department of Cardiology, Shahid Beheshti University of Medical Sciences, Tehran, Iran; ^2^Cardiovascular Research Center, Rajaie Cardiovascular Institute, Tehran, Iran; ^3^Cardiovascular Research Center, Shahid Beheshti University of Medical Sciences, Tehran, Iran

## Abstract

A 73-year-old woman who suffered from major depressive disorder was candidated to receive electroconvulsive therapy (ECT) in combination with medical treatment. Shortly after ECT, she complained of severe chest pain. ECG findings revealed new QRS widening and ST-segment elevation in anterior leads, and echocardiography revealed anteroapical wall akinesia and reduced ejection fraction. Epicardial coronary arteries were normal in emergent coronary angiography, and the diagnosis of Takotsubo cardiomyopathy was considered. In addition, Troponin I was also elevated. The patient had a good recovery after medical treatment for heart failure, and the ejection fraction in echocardiography improved in a few days. This studied case demonstrates the increasing risk of Takotsubo cardiomyopathy following ECT, which needs special attention for patients with chest pain and ECG abnormalities after ECT.

## 1. Introduction

Electroconvulsive therapy (ECT) is an effective and safe treatment used in patients with severe depression and psychosis who are refractory to medical therapy [1]. ECT is a low-risk procedure that can apply to patients with heart diseases [2].

Takotsubo cardiomyopathy (TC), also known as stress cardiomyopathy, is an acute cardiac event that, most of the time, occurs in response to emotional stress. TC mimics an acute myocardial infarction and is presented with chest pain, electrocardiographic and echocardiographic changes, and elevated cardiac markers in the absence of any significant coronary artery stenosis [3] TC constitutes 1%–2% of all patients with acute coronary syndrome and most of the time is a transient condition [4]. TC could occur as a rare complication of ECT and change this safe procedure to a challenging situation. Here, we present a rare case of TC after ECT.

## 2. Case Presentation

The present study case is a 73-year-old woman with recurrent major depressive disorder, which had deteriorated from 2 months ago despite high-dose antidepressant medications. She had also attempted suicide twice during the previous month, so the patient was a candidate for ECT. She was admitted to the psychiatry department of a tertiary referral hospital. She had also received three-session ECT about 15 years ago with dramatic effect without complications. She had no contraindications for ECT.

**Figure 1:**
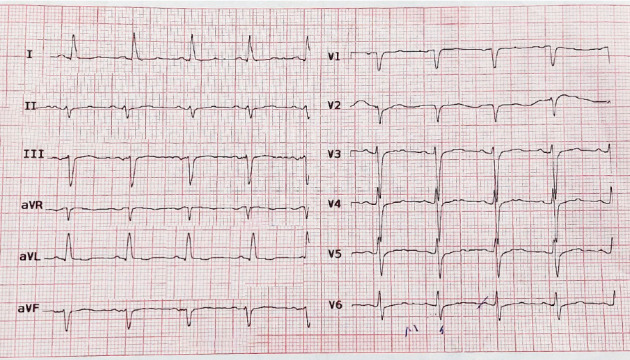
Patient's electrocardiogram at the time of admission.

In the last ECT, her heart rate was 78 beats/min, blood pressure was 120/70 mmHg, axillary temperature was 36°C, and O_2_ saturation was 96%. During the pre-ECT evaluation, an electrocardiogram showed normal sinus rhythm, left axis deviation, and poor R wave progression without any remarkable ST-T changes (Figure 1). Transthoracic echocardiography demonstrated no regional wall motion abnormalities; the left ventricular ejection fraction (LVEF) was 60%.

**Figure 2:**
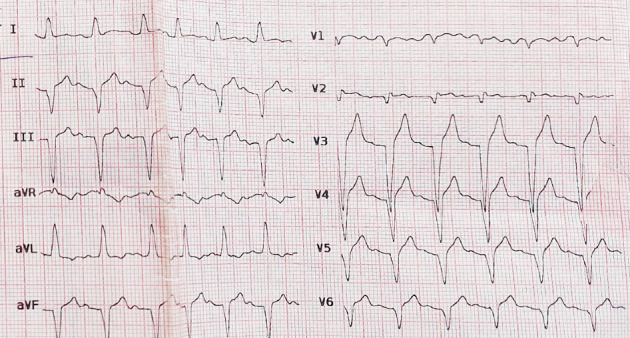
The electrocardiogram taken just after the onset of chest pain shows ST-segment elevation in precordial leads and widening of QRS.

**Figure 3:**
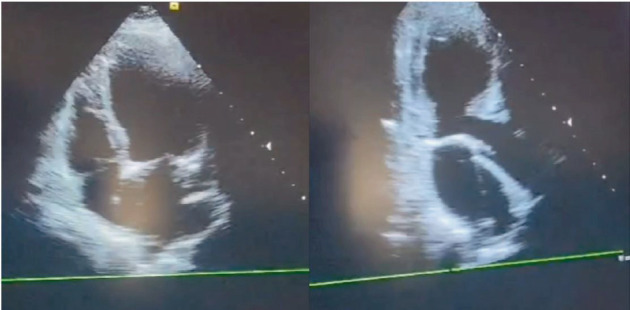
The echocardiogram taken just after the onset of chest pain shows mild left ventricular enlargement, reduced ejection fraction, and akinesia in the anteroseptal and apicoseptal walls.

About 2 h after ECT, she complained of severe retrosternal chest pain and shortness of breath. The electrocardiogram showed a widening QRS and ST-segment elevation in precordial leads (Figure 2). Bedside echocardiogram revealed mild left ventricular (LV) enlargement and severely reduced LVEF (EF: 25%) and anteroseptal and apicoseptal akinesia (Figure 3).

**Figure 4:**
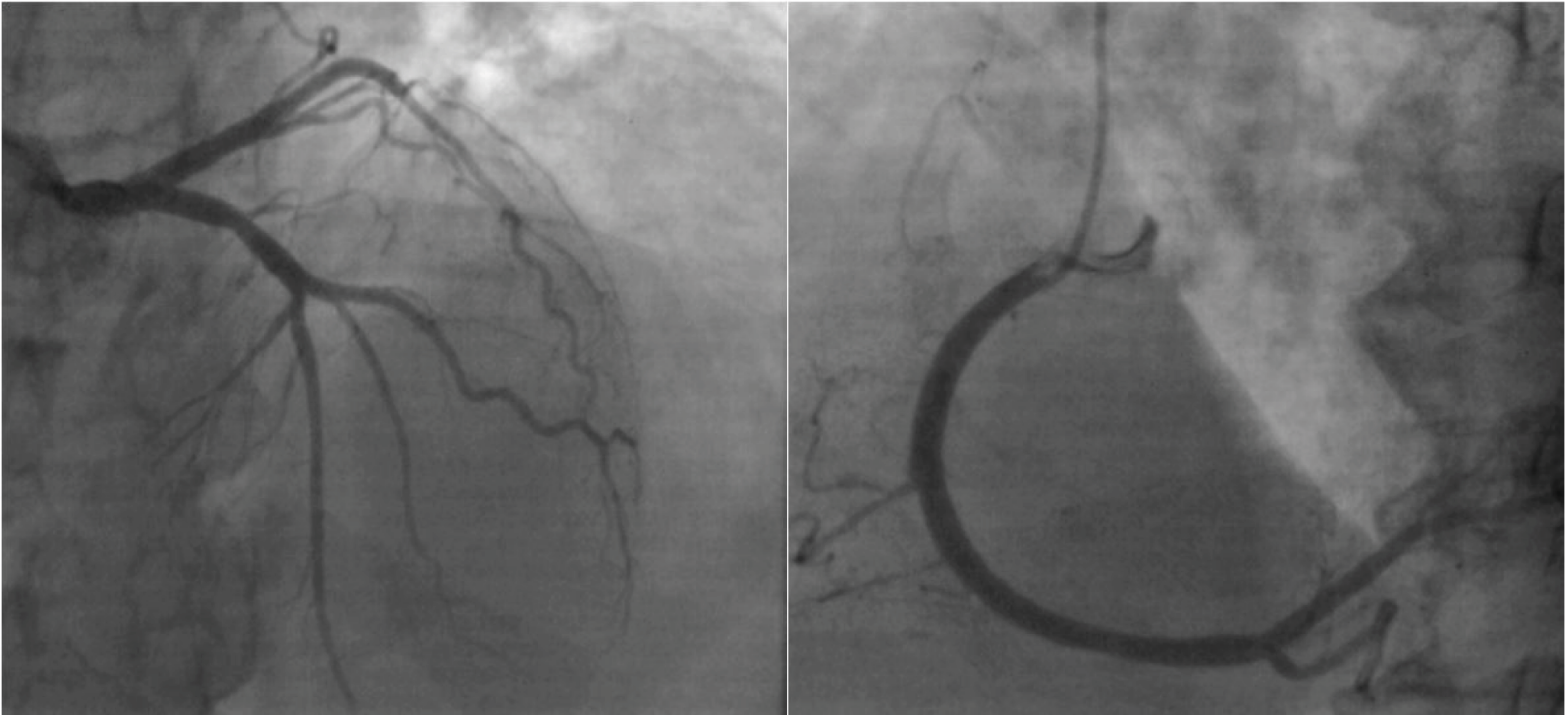
Coronary angiography shows no significant stenosis in the left and right coronary arteries.

She was immediately transferred to the cardiac care unit, and standard treatments of ST-elevation myocardial infarction (STEMI) were initiated. The patient's hemodynamics remained stable (blood pressure: 140/90 mmHg, heart rate: 115 bpm). Then, emergent coronary angiography was performed that showed no significant coronary artery stenosis (Figure 4).

Heart failure management was done with captopril, spironolactone, bisoprolol, and empagliflozin. Troponin levels raised from 0.1 at the onset of chest pain to 1.1 in an hour. Also, CKMB levels rose from 6.2 to 18.4, respectively.

The laboratory results indicated that most parameters were within normal ranges, suggesting stable health markers. White blood cell count, hemoglobin, platelets, sodium, potassium, creatinine, TSH, T3, and T4 were all normal. However, urea was elevated at 57. The urine multidrug screen was negative, showing no drug-related issues.

Conservative treatment continued, and the patient remained stable. Follow-up echocardiography was done after 5 days, and LVEF was increased to 50% with mild anterior hypokinesia. In echocardiography, LVEF was completely normal a week after discharge without regional wall motion abnormality.

## 3. Discussion

This case report demonstrates ECT as a trigger of TC. It is a rare clinical condition that is usually seen in the setting of myocardial infarction.

Acute stress cardiomyopathy, also known as transient LV apical ballooning syndrome or TC, typically involves transient wall motion abnormalities in the LV apex and mid left ventricle. This syndrome occurs in the absence of obstructive epicardial coronary artery disease (CAD) and can mimic STEMI [3–5]. Its incidence is rising to 15–30 cases per 100,000 per year [1]. It is assumed to represent around 1%–2% of all acute coronary syndromes resulting in hospitalization and frequently occurs in post-menopausal women [6–9]. TC is a rare complication of ECT that is reported in some case reports. ECT includes slight electrical stimulation of the brain that causes a generalized tonic–clonic seizure, and it is performed under sedation and a short-acting anesthesia agent. It is used to treat different kinds of psychiatric disorders like major depression, schizophrenia, and bipolar disorder [10]. ECT is prescribed in major depressive or psychotic patients who are treatment-resistant [10, 11], which is known as a highly safe and effective treatment with mild transient complications [1, 12]. The incidence rate of serious complications is low and about 0.097% [13]. TC is a serious complication of ECT and can be predominantly seen in postmenopausal women with depression [6, 14–16], although Medved et al. [6] reported post-ECT cardiomyopathy in a 40-year-old man.

The etiology of stress cardiomyopathy is unclear, but neurally activated or circulating catecholamine-mediated microvascular dysfunction/spasm, myocardial stunning, and injury play essential roles [1, 6, 7]; transient sympathetic release is also seen during seizures in ECT, which causes an increase in heart rate and blood pressure [13].

The proposed diagnostic criteria typically include the presence of transient regional wall motion abnormalities, frequent (but not required) preceding stressful trigger, absence of culprit CAD lesion, abnormal electrocardiographic and biomarker findings, absence of myocarditis or pheochromocytoma, and recovery of ventricular function over subsequent weeks or months. Most patients with stress cardiomyopathy will recover ventricular function rapidly. However, more than 20% of patients do suffer in-hospital complications, including heart failure, arrhythmias, and death in a similar frequency as patients with ACS [4]. Treatment of stress cardiomyopathy is mainly supportive [9]. Sharp and Welch [14] reported that *β*-adrenergic receptor blocking agents could prevent the repetition of stress cardiomyopathy when further ECT is needed. Medwed et al. [6] proposed clozapine use could have a synergic effect with ECT in catecholamine release in TC after ECT. There is a lack of knowledge about the role of SGLT2 inhibitors in TC. Empagliflozin has demonstrated potential in preventing oxidative stress, inflammation, and cardiac remodeling in a rat model of isoprenaline-induced Takotsubo-like syndrome. While it shows promise in mitigating adverse cardiac effects and has been used in other cardiomyopathies, its benefits for Takotsubo patients remain uncertain and require further study [17, 18].

In conclusion, this case report highlights the increasing recognition of TC following ECT, emphasizing further research to understand its pathophysiology and incidence for better prevention and treatment [7]. Additionally, clinicians should be trained to identify high-risk patients, recognize both typical and atypical symptoms of acute coronary syndromes, and be aware of the potential use of ECT after severe complications.

## Data Availability

The data that support the findings of this study are available on request from the corresponding author.
